# A310 CANADIAN GASTROENTEROLOGY RESIDENT ATTITUDES TOWARDS TREATING PATIENTS WITH DISORDERS OF GUT BRAIN INTERACTION

**DOI:** 10.1093/jcag/gwad061.310

**Published:** 2024-02-14

**Authors:** D Rodrigues, C H Parker, M Salim, L W Liu

**Affiliations:** Queen's University, Kingston, ON, Canada; University of Toronto, Toronto, ON, Canada; University of Toronto, Toronto, ON, Canada; University of Toronto, Toronto, ON, Canada

## Abstract

**Background:**

Disorders of gut brain interaction (DGBI) are common in the Canadian population. Studies show DGBI represent 30% of new referrals to gastroenterology (GI) clinics. Despite this, evidence suggests that gastroenterologists experience challenges in providing care for these patients, with a recent study from the United States indicating that this discomfort may begin early in residency training.

**Aims:**

Our aim was to determine Canadian GI resident attitudes towards treating patients with DGBI, with a focus on determining underlying causes and solutions for potential negative attitudes.

**Methods:**

Gastroenterology residents participating in accredited adult GI training programs in Canada were invited to participate in an online 30-item survey. This was based on similar studies in the adjacent field of chronic pain and administered using RedCap. Recruitment occurred through email invites distributed to residency program directors and QR code links presented at national GI resident meetings over one year.

**Results:**

In total, 42 residents participated in the survey. Most residents reported frequent exposure to DGBI. Although most residents felt confident at diagnosing DGBI, only few felt confident in managing them. The majority reported less enjoyment and personal reward at managing DGBI than other gastrointestinal diseases, finding them frustrating to treat and expressing concern regarding the uncertainty of the diagnosis and low likelihood of successful management. Residents believed that challenges in the treatment stemmed from the following: inadequate training, uncertainty regarding the severity of symptoms that were reported, co-existing psychiatric issues, a lack of clarity on therapy and inadequate time. Residents reported that senior residents and staff both expressed negative opinions about caring for those with DGBI, and felt that displeasure for managing patients with DGBI was pervasive in the GI community. Importantly, the majority of trainees believed it was important to formally learn about the physiological underpinnings of DGBI as well as their diagnosis and management. Furthermore, understanding the impact of DGBI on a patient’s quality of life was thought to be important.

**Conclusions:**

This national survey demonstrates that Canadian GI trainees are less confident and less interested in providing care for those with DGBI due to a variety of factors including inadequate training and a potential hidden curriculum. Residents believe in the importance of education in this field. The results of this study will be used to develop educational experiences to ultimately improve the care of those with DGBI.

**Funding Agencies:**

None

